# Fracture Due to Hypercalcemia of Benignancy From a Parotid Tumour

**DOI:** 10.7759/cureus.29446

**Published:** 2022-09-22

**Authors:** Siva Srivastava Garika, Asjad Mahmood, Sai Krishna MLV, Ravi Mittal

**Affiliations:** 1 Orthopaedics, All India Institute of Medical Sciences, New Delhi, New Delhi, IND

**Keywords:** proximal humerus, nonunion, pleomorphic adenoma, hypercalcemia, benignancy

## Abstract

Pseudo-arthrosis of the proximal humerus is an uncommon condition that is difficult to treat. Humoral hypercalcemia from a benign tumour is a rare clinical entity and pleomorphic adenoma as its source has never been reported in the literature. We present the case of a 53-year-old gentleman with a pleomorphic parotid gland adenoma and pseudoarthrosis non-union of the proximal humerus exacerbated by symptomatic parathormone-independent hypercalcemia. The non-union is fixed using a novel technique of placing an ipsilateral cortico-cancellous iliac strut graft as a medial buttress and stabilized with a fixed-angle plate over the lateral side. Following the surgical resection of the tumour, hypercalcemia resolved and the patient improved clinically. This case is a good example of a rare endocrine disease managed by a multidisciplinary approach.

## Introduction

Pseudo-arthrosis of the proximal humerus is an uncommon condition that is difficult to treat [1]. Various fixation methods have been described, along with supplemental bone grafting. We report an unusual case of parathormone (PTH)-independent hypercalcemia of benignancy in which the patient had pleomorphic adenoma of the parotid gland and pseudoarthrosis of the proximal humerus.

## Case presentation

**Figure 1:**
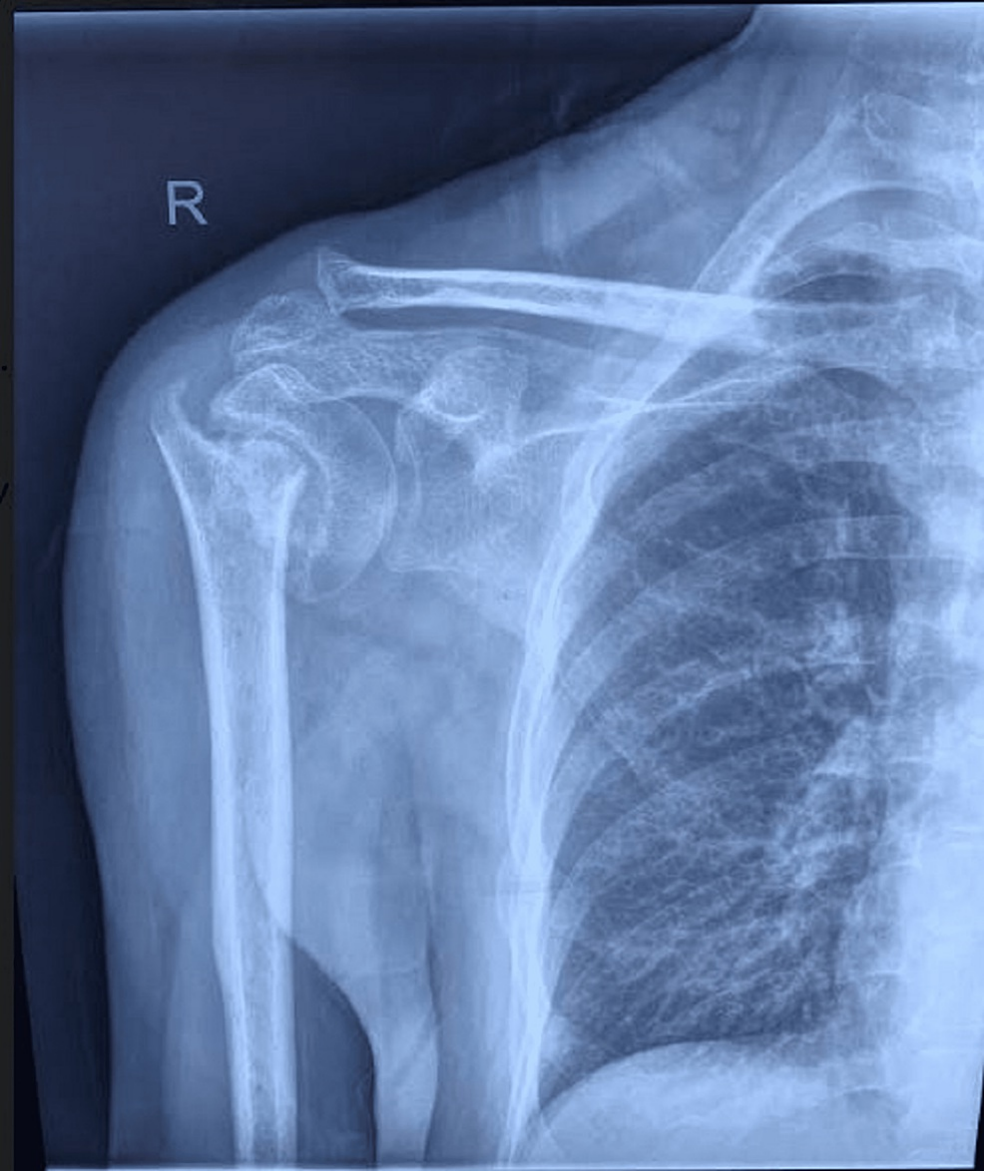
Pre-operative X-ray

A 53-year-old gentleman sustained a right proximal humerus two-part fracture (Figure 1) following a trivial fall three months ago. The patient was managed conservatively for the proximal humerus fracture with U-slab support. The patient did not have any pre-existing comorbidities. He presented to us three months after the injury with wasting of the shoulder girdle muscles with a limited shoulder range of motion. During the evaluation, he had high normal calcium levels, normal PTH levels, elevated levels of alkaline phosphatase (ALP), and low vitamin D levels (Table 1). 

**Table 1:** Biochemistry of the patient during pre-operative period mg/dL, milligrams/decilitre; pg/mL, picograms/millilitre; U/L, units/litre; ng/mL, nanograms/millilitre.

Test	Results	Range
Calcium	10.5 mg/dL	8.6-10.0 mg/dL
Parathormone	18 pg/mL	15-65 pg/mL
Alkaline phosphatase	742 U/L	43-129 U/L
25 hydroxy vitamin D	10 ng/mL	20-50 ng/mL
Phosphorus	3.6 mg/dL	2.5-4.5 mg/dL

**Figure 2:**
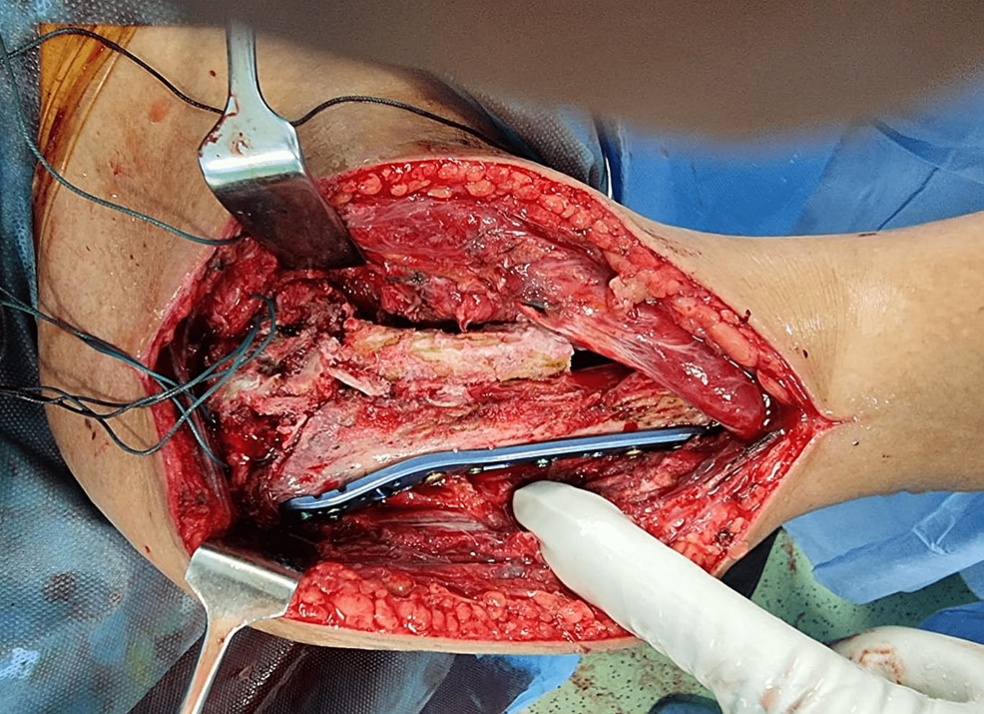
Showing the medially placed iliac bone graft, tension sutures from the rotator cuff, and lateral fixation with a plate.

After an initial evaluation from the endocrinology department, the patient was planned for surgery. The patient was operated on under general anaesthesia using the deltopectoral approach, and the non-union site of the fracture was freshened. Using Ethibond 2 suture bites were taken from the cuff as well as a proximal head fragment and the head was reduced onto the top of the shaft correcting the varus orientation of the head. Dissection was extended on the medial aspect of the fracture below the head and a space was created in the region of the humeral calcar. A 3-5 cm tricortical ipsilateral iliac crest graft was harvested and placed in this space closely abutting the humeral head. The reduction was checked on an image intensifier and temporarily stabilized using wires. A 3-5 cm tricortical ipsilateral iliac crest graft was harvested for bone grafting. A fixed-angle fixation anatomically contoured PHILOS (Proximal Humeral Internal Locking System-AO Synthes) plate was used to fix the fracture and bone graft constructs (Figure 2). The shoulder's stability and range of motion were checked, and the wound closed. A shoulder pouch was given in the immediate post-operative period. Pendulum exercises and shoulder shrugs were started on post-operative day 2.

He was discharged following proper wound care, analgesic management, and a physiotherapy regimen. The patient later presented to the emergency department with complaints of nausea, vomiting, and loss of appetite two weeks following the discharge from the orthopaedic department. His blood biochemistry revealed elevated calcium levels, low PTH, normal Vitamin D, and elevated ALP (Table 2). He was then admitted to the endocrinology department for further evaluation, which revealed hypercalcemia independent of PTH. Initial management with hydration and subcutaneous calcitonin therapies did not lower the calcium levels (refractory hypercalcemia), so a subcutaneous injection of denosumab was administered. Differential diagnoses of hypercalcemia, viz. granulomatous, autoimmune conditions, and multiple myeloma, were evaluated and ruled out. The skeletal survey and serum-free light chain assay were normal; serum and urine protein electrophoresis reported no M spike. A technetium 99m-methyl diphosphonate (MDP) whole-body bone scan suggested no evidence of osteoblastic skeletal metastasis. Then, fluorodeoxyglucose-positron emission tomography (FGD-PET) was done, which revealed metabolically active uptake in the left parotid gland.

**Table 2:** Biochemistry of the patient when presented to the emergency two weeks post-operatively mg/dL, milligrams/decilitre; pg/mL, picograms/millilitre; U/L, units/litre; ng/mL, nanograms/millilitre.

Test	Results	Range
Calcium	14.9 mg/dL	8.6-10.0 mg/dL
Parathormone	4.78 pg/mL	15-65 pg/mL
Alkaline phosphatase	453 U/L	43-129 U/L
25 hydroxy vitamin D	20.9 ng/mL	20-50 ng/mL
Phosphorus	4.2 mg/dL	2.5-4.5 mg/dL

In retrospect, the patient recalled a palpable small non-tender mass over the left parotid region for the last two years, gradually progressive in nature. He then also gave a history of weight loss of nearly 10 to 12 kg over the past two years. Ultrasound neck suggested a solid hypoechoic lobulated lesion in the inferior pole of the left parotid gland. Magnetic resonance imaging reported a left parotid homogenous lobulated mass with homogenous post-contrast enhancement suggestive of a pleomorphic adenoma. The trucut biopsy revealed pleomorphic adenoma, MILAN grade 4A.

The adenoma biopsy specimen was immune positive for the PTH stain. Thus, presuming PTH immunopositivity, which is a marker of PTH-related peptide (PTHrP) secretion, the cause of symptomatic hypercalcemia was probably assumed to be the parotid pleomorphic adenoma. An otolaryngologist consultation was sought and a surgical superficial parotidectomy was done. A specimen of the size of 5 × 4 × 2 cm was resected and sent to confirm the histopathological diagnosis.

In the immediate post-operative period, patients' calcium levels dropped to 6.6 mg/dL (8.6-10 mg/dL), mimicking a hungry bone syndrome, like a scenario that occurs following parathyroidectomy in cases of hyperparathyroidism [2]. The patient was managed with intravenous calcium gluconate in the immediate post--operative period, followed by oral calcium and vitamin D supplements.

**Figure 3:**
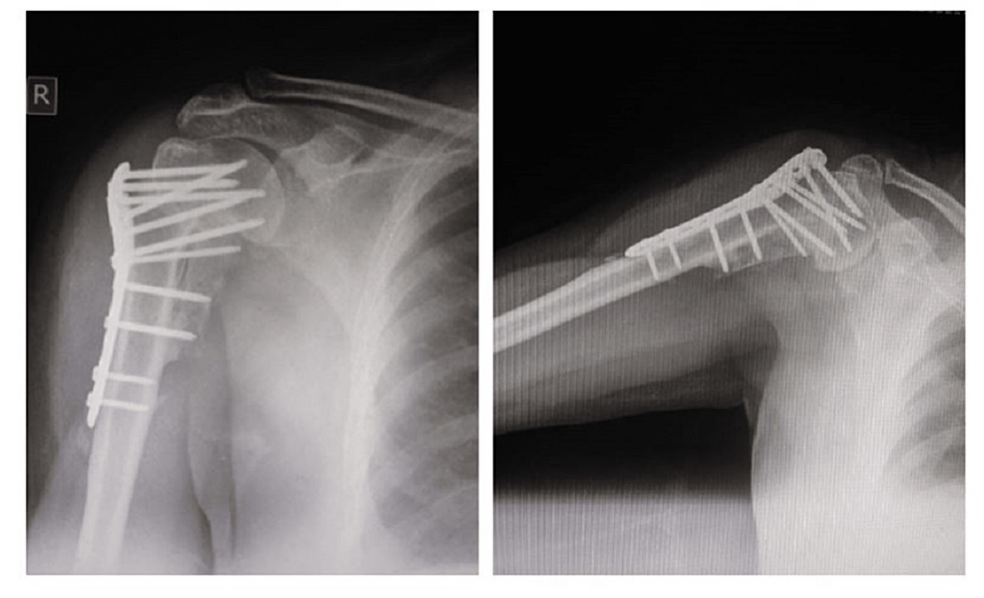
Post-operative X-ray showing union and callus.

**Figure 4:**
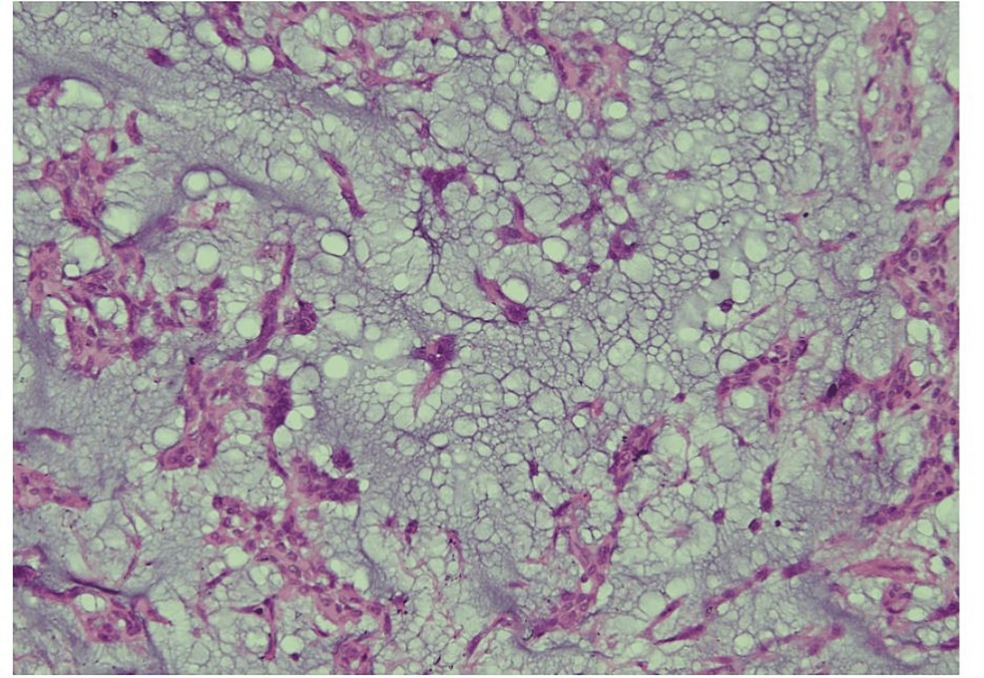
Histopathology shows myoepithelial cells along with tubular and acinar structures in a chondromyxoid background.

**Figure 5:**
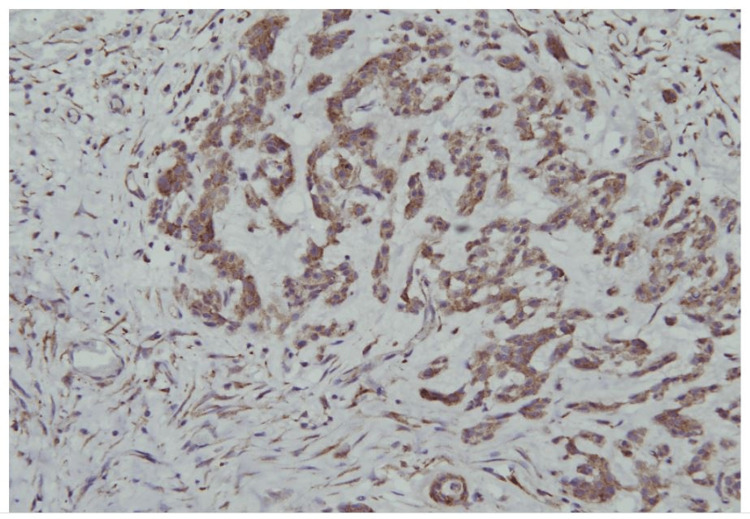
Immunohistochemistry shows diffuse non-homogenous cytoplasmic immunostain in the myoepithelial and epithelial components of the tumour.

During the follow-up period, patients’ calcium levels were normalized, and serum ALP was in a decreasing trend (Table 3). Shoulder rehabilitation involving an active and passive range of motion showed improvement along with the radiological signs of healing (Figure 3). The patient also reported clinical improvement in his general health and weight gain following the tumour resection. Histopathology of the surgically resected specimen revealed pleomorphic adenoma (Figure 4) and the immunohistochemistry was positive for PTH (Figure 5).

**Table 3:** Biochemistry of the patient after pleomorphic adenoma excision mg/dL, milligrams/decilitre; U/L, units/litre.

Test	Results	Range
Calcium	9.1 mg/dL	8.6-10.0 mg/dL
Alkaline phosphatase	233 U/L	43-129 U/L
Phosphorus	2.8 mg/dL	2.5-4.5 mg/dL

## Discussion

The patient presented with a non-union of the proximal humerus with hypercalcemia, probably secondary to humoral hypercalcemia of benignancy from a parotid pleomorphic adenoma.

This is a unique case with a chimaera of entities:

a) pseudoarthrosis of the proximal humerus;

b) humoral hypercalcemia of benignancy; and

c) a benign parotid pleomorphic adenoma as a probable source of secondary hypercalcemia.

The majority of proximal humerus fractures are managed non-surgically (89.1%), with an incidence of 1% to 7% progressing to delayed union or non-union [3].

Non-union originates from either instability or a failure of fracture-healing biology secondary to an infection, inadequate vascularity, bone loss, or a metabolic cause. Elevated parathyroid hormone due to adenoma, hyperplasia, or rarely carcinoma and high calcium levels secondary to increased bone resorption is a known aetiological factor for non-union [4].

Pseudarthrosis has a deranged pathoanatomy and a mortar-and-pestle-like effect which causes cavitation of the head with a shell of bone leading to persistent mobility and making the fixation a challenging task. Medial buttressing, particularly in those with pseudoarthrosis and Boileau type 3 fracture sequelae, is important in achieving a stable construct [5]. Osteosynthesis with bone grafting allows for salvage of the native shoulder joint and has given superior clinical and functional outcomes when compared to arthroplasty [5]. In our case, buttressing was done with an ipsilateral cortico-cancellous iliac strut graft placement medially to counteract the varus force and stabilized with a fixed-angle implant over the lateral side.

In our case, the patient initially had high normal calcium levels, elevated ALP, low normal phosphate levels, low vitamin D levels, and low PTH levels. In 1979, Stewart et al. [6] first used the term "humoral hypercalcemia of malignancy" (HHM) to refer to a malignancy having hypercalcemia without elevated PTH. It is associated with tumour secretion of PTHrP and has lower PTH and vitamin D levels [7,8]. In humoral hypercalcemia, the tumour acts in a paracrine or humoral way, secreting a bone-active substance, most often a PTHrP, in the absence of skeletal metastases resulting in paraneoplastic syndrome. 

PTHrP binds to the common PTH/PTHrP receptors in bone and kidney via its N-terminal domain and mimics PTH action, causing an increase in bone resorption and renal calcium reabsorption, resulting in hypercalcemia [9]. This results in decreased PTH levels. The increase in serum concentration of bone ALP and osteocalcin in these scenarios suggests the humoral agent’s effect on bone, consistent with PTHrP action but not pathognomonic for its action. HHM differs from hyperparathyroidism in terms of its low vitamin D levels, low PTH, and reduced distal tubular calcium reabsorption [8].

Rarely, benign tumours may secrete PTHrP, resulting in symptomatic hypercalcemia presenting with a clinical and biochemical scenario indistinguishable from the HHM, called the humoral hypercalcemia of benignancy [7]. It has been described as a benign tumour of uterine leiomyoma and has also histologically benign lesions of the breast, lymphatics, and adrenal medulla. Thus, humoral hypercalcemia of benignancy is a rare clinical entity to be considered the cause of hypercalcemia [7].

Pleomorphic adenoma is the most commonly occurring benign salivary gland tumour, constituting two-thirds of all salivary gland neoplasms (85%), which occur mostly in parotid glands. In our case, the pleomorphic adenoma was the only suspected cause of hypercalcemia after ruling out all the other differential diagnoses. Based on the clinicopathological assumption, PTH immunostain was sought as a surrogate marker of PTHrP secretion from parotid adenoma and it turned out to be positive. In normal salivary glands, PTHrP is found mainly in the ductal basal and dark cells but not in myoepithelial cells. In pleomorphic adenoma, PTHrP was found in the inner layer of tubuloductal, cyst-like, single-layer ducts, scattered cells, and clusters of squamous metaplasia in pleomorphic adenoma [10]. Thus, PTHrP expression by immunostain is also seen in a normal parotid gland and pleomorphic adenomas not associated with hypercalcemia. Given the clinical presentation of symptomatic hyperparathyroidism, immunopositivity was considered a cause of hypercalcemia of benignancy from a parotid pleomorphic adenoma. With the removal of the tumour, serum calcium levels and serum markers of bone turnover were normalized. The patient's general health also improved.

Although PTHrP is the most commonly detected humoral mediator of hypercalcemia in malignancy, several other mediators have also been implicated such as tumour necrosis factor-alpha, interleukins 1 and 2, transforming growth factor-beta, prostaglandin E2, and granulocyte-macrophage colony-stimulating factor.

## Conclusions

To our knowledge, this rare presentation of non-union pseudoarthrosis of the proximal humerus with probable hypercalcemia of benignancy from a parotid pleomorphic adenoma has not been reported in the literature till now. Although we conclude that the PTHrP from the parotid pleomorphic adenoma may be the cause of hypercalcemia of benignancy in our patient, the complexity of humoral mediators of hypercalcemia prevents us from excluding co-involvement of possible other secreted factors which are to be kept in mind during the management. Hypercalcemia of benignancy is a rare cause to be borne as a differential and a proper whole-body screening is required. This is also an excellent example of a rare endocrine disease managed by a multidisciplinary approach.
